# Self-Sustaining Water Microdroplet Resonators Using 3D-Printed Microfluidics

**DOI:** 10.3390/mi15040423

**Published:** 2024-03-22

**Authors:** Parker A. Awerkamp, David Hill, Davin Fish, Kimi Wright, Brandt Bashaw, Gregory P. Nordin, Ryan M. Camacho

**Affiliations:** Department of Electrical and Computer Engineering, Brigham Young University, Provo, UT 84602, USA

**Keywords:** optics, water, microdroplet, 3D printing

## Abstract

Microdroplet resonators provide an excellent tool for optical studies of water, but water microdroplets are difficult to maintain outside a carefully controlled environment. We present a method for maintaining a water microdroplet resonator on a 3D-printed hydrophobic surface in an ambient environment. The droplet is maintained through a passive microfluidic system that supplies water to the droplet through a vertical channel at a rate equivalent to its evaporation. In this manner, we are able to create and passively maintain water microdroplet resonators with quality factors as high as $3\times 10^{8}$.

## 1. Introduction

Microdroplets have long been used as optical resonators [1]. The surface tension of microdroplets creates a perfectly spherical shape with no surface roughness, making them ideal optical resonators. While many liquids can be used for microdroplet resonators [2], this work focuses specifically on water microdroplet resonators. Water droplet resonators are an ideal tool for biological studies due to the importance of water for life [3,4]. In one study [3], the researchers created a biolaser by suspending bacteria in a microdroplet. These bacteria expressed a fluorescent protein that acted as a gain medium when the droplet was excited with a pump laser. The researchers were even able to create a stable laser using a droplet containing only one bacterium. Such a result would have been extremely difficult to obtain without the use of a water droplet resonator. A solid material would not allow the cells to have sufficient optical interaction with the laser mode, and a water environment was needed to keep the cells alive.

Water droplet resonators have also been shown to enhance our understanding of physical phenomena present in liquids. In one study [5], the researchers were able to characterize capillary waves present on a droplet’s surface by analyzing the droplet’s optical resonance. These angstrom-scale waves have implications in the coalescence of droplets and the spontaneous rupture of liquid films. By using a water microdroplet resonator, the researchers were able to amplify the effects of these capillary waves on the circulating light, providing greater sensitivity in their characterization over other methods.

Additionally, water microdroplet resonators have been used to construct dye lasers [6,7] and Raman lasers [8]. Water resonators also present unique opportunities as a subject of study for tuning resonances [6,9,10] and producing resonators with high quality factors (*Q*) [11,12]. In our work, we present a new platform that will facilitate future studies involving water microdroplet resonators.

Water microdroplet resonators can be challenging to study because of the rapid evaporation of water, which causes the droplet to change size. This effect is especially pronounced at the small scales used for optical resonators. Typical methods to overcome evaporation involve either adding a stabilizer to the water, controlling the droplet environment, or both [13]. Stabilizing agents can be added to water so that the evaporation rate is decreased. This results in the droplet reaching equilibrium at humidities less than 100%. The most common stabilizers are glycerol [3,7,9,12] and salt [10,14]. Glycerol is hygroscopic, allowing it to absorb water from the air to establish equilibrium. Salt lowers the vapor pressure of water with similar results. Alternatively (or additionally), the droplet environment can be controlled by either keeping the droplet in a humidity-controlled chamber [6,8,13] or submerging the droplet in another liquid [15].

Though water naturally forms smooth spherical droplets in a free environment, typically a droplet must be attached to a surface to create a usable optical device. However, surface adhesion can degrade the shape of the droplet for use as a microresonator. This difficulty can be overcome by manipulating the droplet using optical tweezers [14], but ideally the droplet is supported for device functionality and optical interrogation. Past work has demonstrated supporting water microdroplets by placing them on a hydrophobic surface [3,6,7,8,9,10,12,13] or suspending droplets from a glass stem [5,11,16]. These methods, in addition to using optical tweezers, all maintain the droplet in a sufficiently spherical shape to establish optical resonance. The droplets can be formed on these support systems a number of ways, but the most common is to atomize the liquid over a surface or drop an individual droplet onto the support using a pipette or similar tool. Though these methods effectively create droplets of a sufficiently small size (<1 mm), precise control over the size of the droplet is not reported.

We propose a novel solution for droplet formation, support, size control, and stabilization against evaporation. Our design incorporates a microfluidic system that allows us to precisely control the size of the droplet upon formation, which has been used for non-evaporating oil droplets [17] but to our knowledge has not been demonstrated for water microdroplets. Our microfluidic system further serves to provide a constant flow of water to the microdroplet from an external reservoir, completely mitigating the effects of evaporation on the droplet without requiring the addition of a stabilizer to the droplet or a specialized environment. Other methods have been shown to replenish the droplet against evaporation [11], though these did not seek to control droplet size. The microfluidic system we present feeds into a specially designed 3D-printed mount that supports the droplet in a spherical shape and allows for precise droplet positioning. Using this device, we demonstrate water microdroplet resonators that can be tailored to an arbitrary size and are stable against evaporation without requiring additives to the water or a controlled environment.

## 2. Materials and Methods

### 2.1. Device Fabrication

To fabricate the devices, a custom digital light processor stereolithographic (DLP-SLA) printer with a 365 nm LED light source and a pixel pitch of 7.6 μm in the plane of the projected image was used [18]. A custom photopolymerizable resin was used that consists of poly(ethylene glycol)diacrylate (PEGDA, MW258) with a 1% (*w*/*w*) phenylbis(2,4,6-trimethylbenzoyl)phosphine oxide (Irgacure819) photoinitiator and a 2% (*w*/*w*) 2-nitrophenyl phenyl sulfide (NPS) UV absorber [19]. The 3D-printed devices were fabricated on diced and silanized glass slides. Each slide was prepared by cleaning with acetone and isopropyl alcohol (IPA) followed by immersion in 10% 3-(trimethoxysilyl)propyl methacrylate in toluene for 2 h. After silane deposition, the slides were kept in toluene until use. For a typical device, each build layer of the print is exposed to a measured optical irradiance of 21.2 mW/cm^2^ in the image plane for 400 ms, and each layer has a thickness of 10 μm. Adjustments to the irradiance and exposure time were made as necessitated by device geometry. After printing, unpolymerized resin in interior regions was gently flushed with IPA, followed by device optical curing for 30 min in a custom curing station using a 430 nm LED with a measured irradiance of 11.3 mW/cm^2^ in the curing plane.

### 2.2. Design Considerations

**Figure 1:**
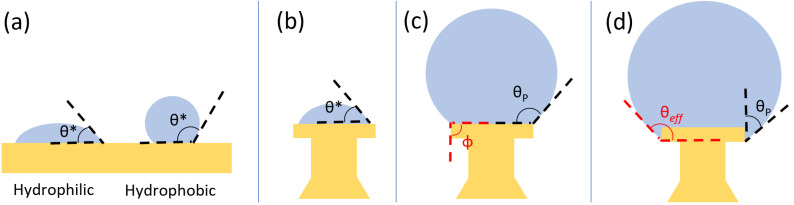
(**a**) Innate contact angle $\theta^{*}$ of a droplet on hydrophilic and hydrophobic surfaces. (**b**) Initially, no corner pinning is present, so the contact angle is $\theta^{*}$. As the drop grows, the liquid becomes pinned (**c**) at the top corner, and the angle formed is $\theta_{P}$ such that $\theta^{*}<\theta_{P}<\theta_{c}$. Further growth causes corner pinning on the bottom edge (**d**), which allows larger droplets to be formed, and the effective contact angle $\theta_{eff}$ becomes $\theta_{P}+90^{{}^{\circ}}$.

The devices were designed to create spherical droplets by leveraging corner pinning. In a system wherein a droplet is supported by a solid surface, the cohesive forces within the droplet and the adhesive forces between the liquid and the solid result in the liquid edge meeting the solid at a given angle, known as the contact angle $\theta^{*}$. As seen in Figure 1a, materials with an innate contact angle $\theta^{*}<90^{{}^{\circ}}$ are considered hydrophilic, while those with $\theta^{*}>90^{{}^{\circ}}$ are hydrophobic. Hydrophobic surfaces are desirable for microdroplet resonators, as their low adhesive forces allow droplets to become nearly spherical due to surface tension; however, by creating structures with corners, we are able to use a hydrophilic material ($\theta^{*}=43^{{}^{\circ}}$) to create our devices. In our devices, as water is added to the top of the device, the contact angle is initially the innate contact angle $\theta^{*}$, as in Figure 1b. To increase the apparent contact angle, we take advantage of corner pinning, as shown in Figure 1c. Corner pinning is a phenomenon in which an advancing liquid cannot proceed around a corner boundary at the native contact angle. Rather, the localized volume of the liquid will increase without advancing around the corner, with the solid–liquid interface increasing in angle until the critical angle $\theta_{c}$ is reached, where $\theta_{c}=\left(180-\phi +\theta^{*}\right)$ and ϕ is the angle of the corner boundary relative to a flat surface. The result is a pinned contact angle $\theta_{P}$, where $\theta^{*}<\theta_{P}<\theta_{c}$, which allows us to create spherical droplets on surfaces that are innately hydrophilic by supporting the droplets on posts with corner boundaries. Our design further leverages corner pinning using a re-entrant structure, wherein the undercutting of the head creates an additional corner boundary. If the droplet advances from the top of the device and along its side, the droplet pins on the bottom edge, creating an effective contact angle $\theta_{eff}=\theta_{P}+90^{{}^{\circ}}$ (see Figure 1d).

**Figure 2:**
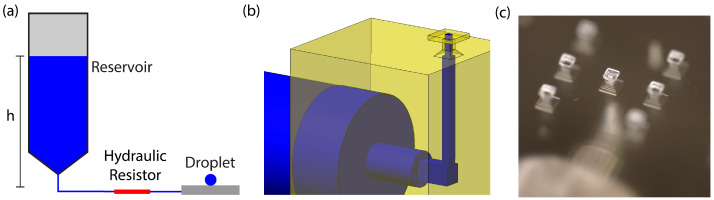
Microfluidic system of the devices. (**a**) The pressure at the hydraulic resistor is determined by the height *h* of the water reservoir (which is open to the air). This pressure results in constant flow through the resistor, matching the evaporation rate of the droplet. (**b**) Model of the 3D-printed device. The device has a channel to allow water flow and a head with a re-entrant shape to create the corner pinning needed for a spherical droplet. (**c**) Microscope image of the 3D-printed device.

This re-entrant design allows the head to support larger droplets and increase the effective contact angle from the innate $\theta^{*}=43^{{}^{\circ}}$ to the effective contact angle $\theta_{eff}=130^{{}^{\circ}}$, which helps to create more spherical droplets. Figure 2b,c show a model and microscope image of our device, respectively. Our device’s dimensions are based on the fact that the 3D printer has a pixel size of 7.6 μm and a layer thickness of 10 μm. As such, all dimensions are designed to be multiples of this pixel size, and we provide the design parameters here. The device consists of a chamfered square base with side length 176 μm and a square pillar 50 μm tall and 76 μm wide. These are topped with a square head that is 106.4 μm wide and 10 μm thick. Water is supplied to the top of the head by a square channel 45.6 μm wide that cuts through the head, column, and base. The channel connects to larger openings that accommodate external tubing. The device was designed to accommodate as small a structure as possible while ensuring the device could be reliably fabricated.

### 2.3. Microfluidic System

The microfluidic system (shown in Figure 2a) functions by providing a constant flow of water to the droplet such that the rate of evaporation from the droplet is matched by the rate of water flowing into the droplet. The flow rate into the droplet can be precisely determined and set by use of our microfluidic system, which was designed on the principle of hydraulic resistance [20]. We here present a theoretical mathematical description of the process.

The hydraulic resistance of a microfluidic channel is given by analysis of the Hagen–Poiseuille law, resulting in

(1) $$R_{h}=\frac{8\mu L}{\pi {\left(r_{h}\right)}^{4}}$$

where $R_{h}$ is the hydraulic resistance, μ is the viscosity of the liquid, *L* is the length of the channel, and $r_{h}$ is the channel radius for a cylindrical channel. The effective $r_{h}$ for other channel geometries can be calculated as $r_{h}=2A_{cs}/P_{cs}$, with $A_{cs}$ and $P_{cs}$ being the cross-sectional area and perimeter of the channel, respectively. The hydraulic resistance of a channel can be used to determine the flow rate of water (or any liquid) in the channel through the equation

(2) $$F=\frac{\Delta P}{R_{h}}$$

where *F* is the flow rate, $R_{h}$ is the hydraulic resistance of the channel, and ΔP is the difference in pressure across the channel. This relation is similar to Ohm’s law for electric circuits and allows us to analyze a microfluidic system by comparing or combining the hydraulic resistances of various microfluidic components as needed. Our system takes advantage of this by incorporating a hydraulic resistor [20].

The hydraulic resistor serves two purposes. The first is to dominate the overall hydraulic resistance of the system. In our system, the hydraulic resistor consists of a 1 cm long fused silica tube with an inner diameter of 25 μm, giving it a resistance 1000 times greater than the combined resistance of all other microfluidic elements in our system (tubing, 3D-printed channels, etc.). In this way, any variation between devices is negligible, and calculations can safely neglect other microfluidic elements. The second purpose of the hydraulic resistor is to match the desired flow rate to an experimentally convenient reservoir depth (approximately 10 cm).

To match the evaporation rate to the flow rate into the droplet, the height of the water in the reservoir is adjusted. This correct height is calculated by comparing the rate of evaporation with the flow rate of the microfluidic system. The flow rate *F* of the hydraulic resistor (which dominates the system) is given by Equation (2). The pressure *P* at each end of the resistor is determined by the height of the water above it as given by P=ρgh, where ρ is the density of water, *g* is the acceleration due to gravity, and *h* is the water height. Thus, the pressure difference across the resistor is ΔP=ρgΔh, where Δh is the height difference between the height of the reservoir and the droplet.

The droplet evaporation rate *E* can be found by analysis [21] of Fick’s law, resulting in the relation

(3) $$E\approx 4\pi rD(c_{0}-c_{\infty })$$

where *r* is the radius of the droplet, *D* is the diffusion coefficient of water vapor in air, and $c_{0}$ and $c_{\infty }$ are the vapor density at the droplet surface and infinity, respectively. This evaporation rate can be combined with the previous equations to determine the height needed to maintain a droplet of a given radius *r* through

(4) $$h\approx R_{h}\frac{4\pi D(c_{0}-c_{\infty })}{\rho g}r$$

which, for a droplet of radius r=250 μm and typical lab conditions (21 °C, 50% humidity), equates to a height of h=6.5 cm.

The relationship between height and droplet radius are strongly dependent upon the environmental conditions, particularly the temperature and humidity. Furthermore, the model described above represents a simple explanation of the system, particularly for determining the evaporation rate. In practice, other effects (such as airflow) contribute to evaporation but are not addressed in this explanation. Therefore, while it is very difficult to determine mathematically what the diameter of a stable droplet will be based on the height of the reservoir, through experimentation we determined that the water height needed to maintain a droplet at various temperature and humidity values remains in a range that we can address with our setup. For reasonable lab conditions, a droplet with r=250 μm can be maintained at water heights ranging from 2 cm to 20 cm.

### 2.4. Experimental Procedure

**Figure 3:**
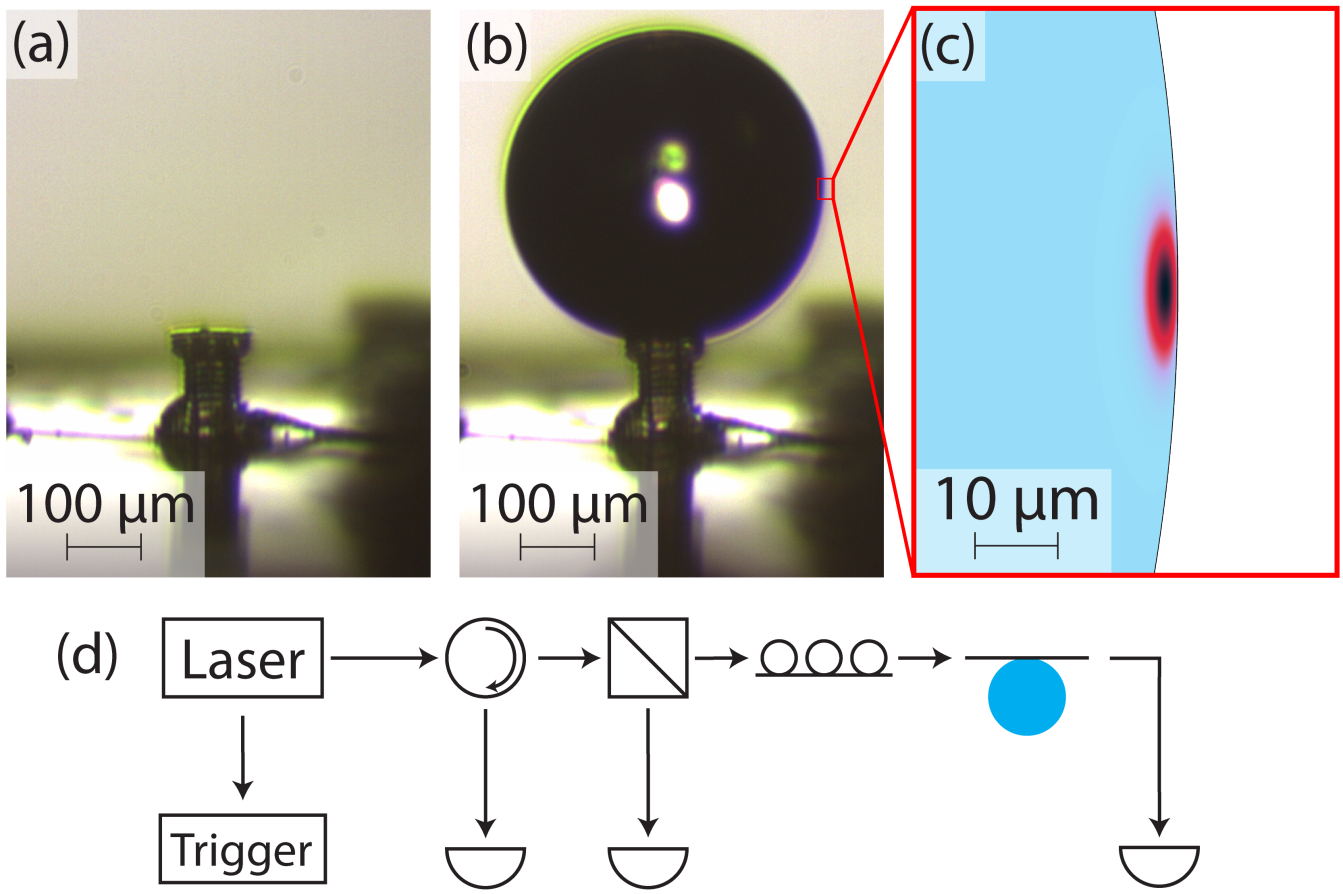
(**a**) The 3D-printed device for supporting microdroplet resonators. (**b**) Water droplet supported by the 3D-printed device. (**c**) Fundamental Z polarized mode of the droplet. Water is shown in light blue, and air is shown in white. (**d**) Visualization of the optical path. Light from a laser passes through a circulator, then a beamsplitter, then a polarization controller. The light is then coupled to the droplet via a tapered fiber.

The experimental procedure is outlined in Figure 3. The device before a droplet is formed is shown in Figure 3a. Figure 3b shows a droplet on the device head, with corner pinning on the top edge evident. A droplet is initially formed by applying a small amount of external pressure (less than 1 psi) to the water reservoir. After the droplet is formed, the external pressure is turned off, and a valve is opened to ensure the reservoir remains at atmospheric pressure for the remainder of the experiment. At this point, the flow rate of water to the droplet is entirely determined by the reservoir height.

In practice, it is often more intuitive to consider matching the flow rate into the droplet with the evaporation rate of the droplet rather than considering that the size of the droplet is determined by the reservoir height. The reservoir height is proportional to the flow rate, and the evaporation rate of a droplet of a given size is constant. If the flow rate is greater than the evaporation rate, the droplet grows, and if the flow rate is lower than the evaporation rate, the droplet shrinks. With this in mind, in our experiments we form a droplet and allow it to expand until it is near the desired size. We then adjust the height of the water reservoir to fine-tune the droplet size and find equilibrium: the point at which the droplet has the desired size and no longer grows or shrinks. In our experience, this fine-tuning is vital for determining the required reservoir height for a droplet of a given size.

This procedure may be aided by the naturally stabilizing action of droplet evaporation, since the droplet evaporation rate is proportional to the droplet size (see Equation (3)). If we assume the flow rate into the droplet is larger than the evaporation rate, the droplet will grow in size. However, as the droplet grows, the evaporation rate also increases. This continues until the evaporation rate matches the input flow rate, and the droplet stabilizes. The same effect occurs for a shrinking droplet. In this way, we can adjust the reservoir height until the droplet is as stable as possible at the desired size, and then small mismatches in the flow rate and evaporation rate of the droplet will naturally stabilize. However, as explained previously, the theoretical model does not account for all possible system dynamics. We have often seen in our experiments that droplets tend to exhibit some form of hysteresis in that growing droplets tend to keep growing if left alone and shrinking droplets tend to continue shrinking if left alone. The most reliable method for forming a stable droplet is to actively tune the reservoir height until the droplet becomes stable.

This procedure leads to stable droplets. In our context, the droplet being stable means that its size is either constant or that changes to the size are gradual enough that optical coupling is still possible for an extended time. In our experiments, we are able to optically couple the droplet resonator to the tapered fiber for over a minute. As the coupling distance is less than 1 μm, the droplet size therefore changes at a rate less than 1 μm/min. In one experiment, the droplet size was measured and changed in diameter from 524 μm to 518 μm over the course of 15 min, giving dr/dt=3.3 nm/s. In comparison, a droplet placed on a similar head but without our microfluidic system changed in diameter from 867 μm to 559 μm over the course of 6 min and had completely evaporated 6 min after that. This results in dr/dt=431 nm/s.

Once the droplet is stable, the droplet is brought into close proximity with a tapered optical fiber. The fiber is tapered using a heat-and-pull rig similar to those used in [22,23,24,25]. After it is tapered, the fiber is treated with Sigmacote (SL2, Millipore Sigma, Darmstadt, Germany), giving it a hydrophobic coat. This hydrophobic coat makes coupling to the fiber much easier. Without the coating, there is often an attractive force between the fiber and the droplet, causing them to touch when brought close together. Upon touching, the droplet deforms, eliminating resonance, and can leave residue on the fiber. The coated fibers are able to make slight contact with the droplets without destroying the resonance, and they prevent residue from being left on the fiber, allowing for repeated experiments on the same fiber. To the authors’ knowledge, this is the first use of a hydrophobic coating on a tapered fiber to counteract attractive van der Waals forces, and it significantly reduces the difficulty of optical coupling.

The optical setup of our experiment is shown in Figure 3d. A wavelength-tunable laser (Sachertek PZ500, Sacher Lasertechnik, Marburg, Germany) is swept repeatedly near 930 nm, and a trigger signal is sent from the laser to an oscilloscope to correlate the wavelength with the measured optical signals. The light is sent through a circulator, with the return port being monitored for backscattering, then through a beamsplitter. The beamsplitter provides a reference signal that is used in postprocessing to normalize the variations in the power output of the laser due to the wavelength sweep. The signal beam continues to polarization paddles and finally to the tapered optical fiber, which is evanescently coupled to the water droplet. The transmission of the laser through the tapered fiber is monitored as the droplet is brought closer to the fiber until resonant features appear. Many resonant modes can be excited in this way, and Figure 3c shows the simulated fundamental mode.

## 3. Results

Using the methods described above, we are able to create stable water microdroplets. The device geometry limits the size of droplets to diameters between 300 μm and 600 μm, as excessively small droplets do not exhibit a spherical shape, and large droplets advance beyond the corner boundaries of the devices and collapse. In one experiment, we created stable droplets with diameters of 400 μm, 500 μm, and 600 μm. We measured the reservoir height for each and compared to the mathematically expected heights. We found that, in practice, the measured heights were significantly larger than expected, but that the change in reservoir height needed to create droplets of a different size was the same. That is, the reservoir height needed experimentally had a constant offset from the calculated value, as shown in Table 1. We believe this is due to a higher evaporation rate. This difference could be caused by air currents or other dynamics. It has been shown that the evaporation rate of sufficiently small droplets can be more complex than the treatment we provide here [26,27].

**Figure 4:**
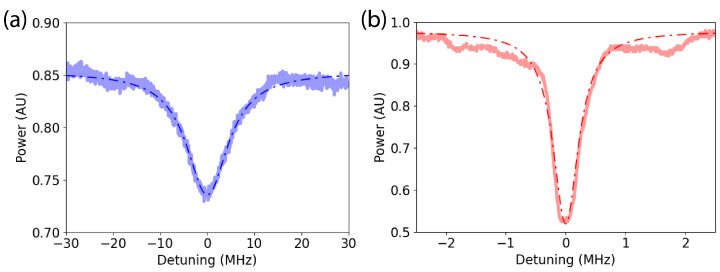
Resonant features of water microdroplets. The solid lines show the measured optical power, and the dashed lines show a fit to a Lorentzian. (**a**) A peak with $Q=3.01\times 10^{7}$. (**b**) A peak with $Q=6.25\times 10^{8}$.

We coupled light into the droplets as described previously. The resulting data exhibit resonant peaks with a high quality factor (*Q*). Two peaks are shown in Figure 4, wherein the measured optical power is shown with a solid line and the fitted Lorentzian is shown with a dashed line. Prior to fitting, the data were normalized to remove the wavelength dependence of the rest of the optical system by dividing the throughput power by the reference signal and normalizing to the maximum power.

The peaks in these plots were fitted to a Lorentzian by using a rate equation model [28]. After fitting the peaks, we found their quality factor (*Q*) through linewidth analysis. The peaks shown in Figure 4 were found to have $Q=3.01\times 10^{7}$ (part (a), shown in blue) and $Q=6.25\times 10^{8}$ (part (b), shown in red). Many other peaks were analyzed and similarly demonstrate *Q* values on the order of 10^7^–10^8^.

## 4. Discussion

The quality factors reported in the previous section were exceptionally high and merit additional discussion. The overall *Q* of a microdroplet resonator can be expressed as a combination of contributing *Q* factors associated with various loss mechanisms. As such, we can express the overall *Q* as [29]

(5) $$Q^{-1}=Q_{ab}^{-1}+Q_{s}^{-1}+Q_{rad}^{-1}$$

where $Q_{ab}$ is the *Q* related to material absorption, $Q_{s}$ is the *Q* related to surface scattering, and $Q_{rad}$ is the *Q* related to radiative losses. Based on this relationship, the maximum value of *Q* is limited to the lowest of the contributions from loss mechanisms. In our devices, the curvature is relatively large and the surface is extremely smooth, so we expect both $Q_{rad}$ and $Q_{s}$ to be very large. As such, we expect $Q_{ab}$ to limit the total *Q*. The material absorption *Q* can be calculated by $Q_{ab}=2\pi n/\alpha \lambda$, where n=1.3278 [30] is the refractive index of water, α = 0.20365 cm^−1^ [30] is the absorption coefficient of water, and λ = 930 nm is the free-space wavelength of the light. The result is $Q_{ab}=4.4\times 10^{5}$, and we would expect this to be the maximum possible value for the *Q* of a water resonator. However, in our experiments, we measure *Q* values much higher than this.

We have explored several explanations for this discrepancy, including thermal bistability, systematic errors in data collection, and dynamic droplet sizes during measurement. Thermal bistability is caused when the intensity of the light in the droplet causes heating to occur, which causes a change in the refractive index of the liquid. This causes a shift in the resonant wavelength of the resonator and can be seen as a widening or narrowing of the resonant peak as the laser sweeps. Depending on the relative directions of the laser wavelength sweep and the resonance shift, the peak may appear narrower or broader than its natural linewidth. Since water has a negative thermo–optic coefficient (increasing temperature leads to decreasing refractive index), we would expect to see linewidth broadening while scanning toward shorter wavelengths and linewidth narrowing when scanning toward longer wavelengths [11]. We conducted experiments wherein the wavelength was swept from 930 nm to 931 nm and back, capturing both sweep directions. No significant change in *Q* was observed between the sweep directions, and, as such, we do not believe thermal bistability to be the cause of the higher-than-expected *Q*.

We considered instability in droplet size to be another likely reason for measuring a higher *Q* than expected. Though the droplets are very stable, subtle growth or shrinkage of the droplet has been observed. In particular, droplets that are growing slightly are commonly seen, as droplet growth aids with reducing the gap between the droplet and the tapered fiber, allowing for coupling. The effect of a growing droplet on the quality factor of the resonator is expected to be similar to that of thermal broadening and narrowing, as a changing droplet size also will result in a moving resonant wavelength. As such, we would expect to see a difference in the *Q* of resonances measured with a low-to-high wavelength sweep versus a high-to-low wavelength sweep. As stated above, no significant difference was observed for such sweeps.

Another possibility is that the resonant wavelength is changing so fast due to instability that the wavelength of the laser is effectively stationary in comparison. In this case, the direction of the laser sweep would be irrelevant. For this to be the case, the droplet’s resonant wavelength ($\lambda_{0}$) would need to be changing at a rate such that the difference in the actual ($Q_{act}$) and measured ($Q_{meas}$) quality factors of the resonator and the sweep speed of the laser ($d\lambda_{las}$) and the rate of change of the resonant frequency ($d\lambda_{0}/dt$) are the same; that is

(6) $$\frac{d\lambda_{0}/dt}{d\lambda_{las}/dt}=\frac{Q_{meas}}{Q_{act}}$$

since a faster relative change in wavelength between $\lambda_{0}$ and $\lambda_{las}$ results in a higher measured *Q*. For the blue peak shown in Figure 4a, the laser sweep speed was $d\lambda_{las}/dt$ = 1 nm/s, and the measured *Q* was $Q_{meas}=3.0\times 10^{7}$. Using the absorption-limited *Q* as a maximum value for the actual *Q*, we get $Q_{act}=Q_{ab}=4.4\times 10^{5}$. Using these values with Equation (6), we find $d\lambda_{0}/dt=68$ nm/s. This represents a conservative estimate of $d\lambda_{0}/dt$, as either a higher measured *Q* (such as the red peak in Figure 4b) or a lower $Q_{act}$ (if we considered loss mechanisms other than absorption) would result in a higher $d\lambda_{0}/dt$.

Using $d\lambda_{0}/dt$, we can find the expected rate of change of the droplet radius dr/dt by taking the derivative of the equation $\lambda_{0}=2\pi rn/m$, resulting in

(7) $$\frac{dr}{dt}=\frac{d\lambda_{0}}{dt}\frac{m}{2\pi n}$$

where *r* is the radius of the droplet, *n* is the refractive index of water, and *m* is the azimuthal mode number. Using m=2230 (which corresponds to r=250.005 μm at $\lambda_{0}=930$ nm), we calculate dr/dt=20.165 μm/s. This is an unrealistically high rate for a droplet that appears to be stable. If the droplet were growing at this rate, the radius would double in 12.5 s, which was not observed in our experiments. As previously discussed, stable droplets have a radius rate of change of under 1 μm/min, and a typical droplet was measured to have dr/dt=3.3 nm/s.

Finally, we investigated our data collection and analysis process to ensure we were accurately correlating our measurements to the laser wavelength, fitting the resonant peaks, and calculating the *Q*. We found our process to be robust and our analysis to be accurate. In the course of our study, we performed experiments using different components (fiber, 3D-printed device, and hydraulic resistor), and we see similarly high values in all cases. As such, though we do not fully understand the cause of these high *Q* values, we believe them to be accurate. These results merit further study and replication by future researchers, as the *Q* values we report are significantly higher than any previously reported quality factors for water resonators.

## 5. Conclusions

In conclusion, we have demonstrated a system that uses 3D-printed microfluidic mounts to create stable water microdroplet resonators in ambient environments. The system we present overcomes the effect of droplet evaporation by supplying a constant flow into the droplet in stable equilibrium. The resulting resonators exhibit very high optical quality factors. These results may lead to future systems that integrate additional microfluidic technologies such as mixers and valves [18]. Integrating such microfluidic devices to the microresonator mount could allow for real-time manipulation of the droplet’s contents for mixing and adding analytes or dyes on demand. The system we present here provides a basis for such future work and demonstrates a unique solution for the challenges inherent in using water micodroplet resonators.

## Figures and Tables

**Table 1:** Experimental and calculated reservoir heights and the differences between them for given resonator sizes.

Droplet Diameter [μm]	Reservoir Height (Experimental) [mm]	Reservoir Height (Calculated) [mm]	Difference [mm]
400	147	40	107
500	155	50	105
600	165	60	105

## Data Availability

The data presented in this study are openly available in FigShare at http://doi.org/10.6084/m9.figshare.24556363 (accessed on 19 March 2024).
